# An Analytical Modeling Study on the Thermal Behavior of Copper–Carbon Nanotube Composite Through-Silicon Via (TSV)

**DOI:** 10.3390/nano16060377

**Published:** 2026-03-21

**Authors:** Kai Ying, Jie Liang

**Affiliations:** The School of Micro Electronics, Shanghai University, Shanghai 201800, China; 23723761yingkai@shu.edu.cn

**Keywords:** copper–CNT (Cu-CNT) composite, through-silicon via (TSV), analytical model, thermal conductivity

## Abstract

In this study, the Monte Carlo (MC) method is employed to generate the diameter and relative positional distributions of carbon nanotubes (CNTs). Based on this, we develop a three-layer thermal model for a copper-carbon nanotube (Cu-CNT) through-silicon via (TSV). By integrating Gauss–Hermite quadrature with the Law of Large Numbers (LLN), an analytical expression for thermal conductivity is derived, enabling efficient and accurate estimation of the thermal conductivity of Cu-CNT-filled TSV. Contrary to expectations, the thermal conductivity of TSV does not increase significantly with CNT volume fraction, primarily due to the interfacial thermal resistance at Cu-CNT and CNT-CNT junctions. Through calibration against previously reported experimental data, the effective Cu-CNT interfacial thermal resistance is estimated to be on the order of 10^−7^ m^2^K/W. Comparison with previously reported effective thermal conductivity data of Cu-CNT composites shows that the model maintains an error below 2% when the CNT volume fraction is below 10%. The model is therefore most suitable for low CNT volume fractions, where the assumed spatial distribution and structural simplifications remain physically valid. Furthermore, this study investigates the influence of TSV length on thermal performance, predicts the variation in thermal conductivity of Cu-CNT composites under different volume fractions, and the extracted thermal conductivity values are further used as material inputs for device-level electro-thermal COMSOL 6.1 simulations.

## 1. Introduction

Three-dimensional integrated circuits (3DICs) are considered a promising candidate technology for overcoming the limitations of Moore’s Law. By leveraging vertical space more efficiently, 3DICs offer advantages such as high-density stacking, lower power consumption, and enhanced performance [1]. As a key enabling technology for 3DIC integration, through-silicon vias provide vertical interconnections for multi-chip stacking systems, ensuring efficient data transmission while improving overall integration density [2].

The development of conventional copper (Cu)-filled TSV technology has significantly increased bandwidth, reduced signal delay, and optimized power management in various applications. However, due to high current densities, the electromigration of copper atoms poses substantial reliability challenges [3,4,5]. Carbon nanotubes (CNTs), with their ultra-high current-carrying capacity (1 × 10^9^ A/cm^2^) [6], exceptional mechanical properties, and excellent electromigration resistance [7], have been proposed as a potential replacement for Cu in interconnect materials.

An alternative and promising solution lies in the development of copper-carbon nanotube (Cu-CNT) composites. Cu-CNT composites exhibit outstanding electrical and thermal properties, making them highly promising for next-generation interconnect applications. Electrically, they possess excellent conductivity (2.3–4.7 × 10^5^ S/cm^−1^) and an ultra-high current-carrying capacity (6 × 10^8^ A/cm^−2^) [8]. Thermally, they combine high metallic thermal conductivity (approximately 395 W/m·K) with a coefficient of thermal expansion (CTE) similar to that of silicon (5.0 ppm/K) [9]. Although previous studies have explored Cu-CNT TSV models by separately analyzing copper and CNTs [10,11], they have largely overlooked the complex spatial distribution of CNTs within TSV, which poses challenges for design compatibility with manufacturing processes. Moreover, most Cu-CNT TSV models remain confined to numerical solutions, often involving computationally intensive procedures.

In this work, we employ the Monte Carlo (MC) method combined with a Gaussian distribution to simulate the spatial distribution of CNTs in a more realistic manner. Particularly, by integrating Gauss–Hermite quadrature and the Law of Large Numbers, we significantly enhance computational accuracy and efficiency compared to traditional numerical methods. We establish an analytical thermal model for Cu-CNT TSVs and propose a formula for their thermal conductivity.

Accurate modeling of thermal conductivity in Cu-CNT TSVs is crucial for enhancing reliability and thermal management in practical 3DIC applications. Based on this analytical model, we comprehensively evaluate the feasibility of Cu-CNT TSVs, analyze Cu-CNT interfacial thermal resistance issues, and further investigate thermal simulations and reliability aspects. While the current analytical model demonstrates promising accuracy, further optimization of parameters and broader validation under different fabrication conditions remain necessary.

## 2. Equivalent Thermal Modeling for Cu-CNT TSVs

### 2.1. CNT Distribution

Building upon the MC concept and optimizing the methodology from [12], we simulate the spatial distribution of CNTs within the TSV.

(1) Random numbers are generated such that CNT positions follow a normal distribution, while their diameters follow a log-normal distribution [13]. This simulation approach aligns with experimental observations of CNT growth, enabling a realistic representation of the CNT distribution in the TSV [14].
(2) The CNT distribution is constrained within the circular cross-section of the TSV, ensuring that the distance between any two CNTs is greater than the minimum separation distance d. Regions not occupied by CNTs are filled with Cu.

**Figure 1:**
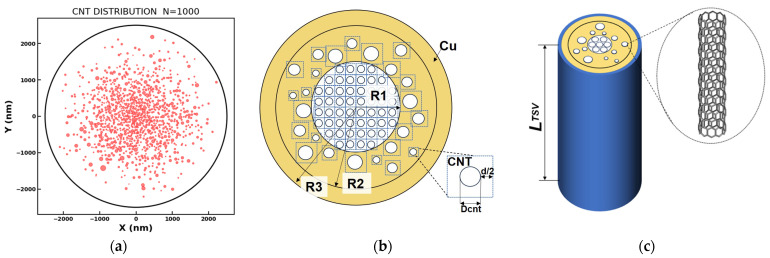
(**a**) Distribution results obtained by the MC method with 1000 samples. (**b**) Schematic of the three-layer Cu-CNT TSV model. (**c**) 3D schematic of the three-layer Cu-CNT TSV model. The red dots represent the positions of CNTs, which follow a normal distribution. *R*_1_, *R*_2_, and *R*_3_ are the radii defining boundaries of innermost, middle, and outermost layers, respectively. *r*_*i*_ is the radius of an individual CNT, which follows a log-normal distribution characterized by mean *μ*_D_ and standard deviation *σ*_D_. *d* is the minimum separation distance between CNTs.

As shown in Figure 1a, we randomly generated 1000 red circles based on the Monte Carlo (MC) method to represent the actual spatial distribution of CNTs within the TSV. The XY coordinates in Figure 1a are used solely to indicate the relative positions in the model. Considering the van der Waals interactions between adjacent CNTs, the minimum separation distance is set to *d* = 0.34 nm. According to experimental findings on Cu-CNT composite growth, CNTs exhibit a higher density near the center of the TSV. Therefore, a Gaussian distribution is adopted in this study to describe the spatial distribution of CNTs [14]. In addition, the CNT diameters are assumed to follow a log-normal distribution, a widely validated assumption supported by numerous CNT growth experiments [13]. This probabilistic distribution model can be adjusted according to actual CNT experimental data, providing excellent adaptability for various practical applications.

### 2.2. Analytical Model of Cu-CNT TSV

Carbon nanotubes can be classified into single-walled carbon nanotubes (SWCNTs) and multi-walled carbon nanotubes (MWCNTs) based on the number of concentric graphene layers. In this study, we assume that the CNTs used are predominantly MWCNTs, which aligns with practical TSV applications [15]. Figure 1b shows a schematic of the three-layer Cu-CNT TSV model. Based on Figure 1a, which shows the simulated distribution results, we approximate the Cu-CNT TSV model as follows: each CNT, along with a surrounding region extending radially outward by a distance of *d*/2 from its center, is treated as an individual unit. We further assume that in the inner cylindrical region (*r* ≤ *R*_1_), CNTs are densely packed with uniform spacing equal to the minimum separation distance *d*. In contrast, within the outermost annular region (*R*_2_ < *r* ≤ *R*_3_), the CNT density is sufficiently low; thus, we approximate this region as fully occupied by Cu, neglecting the presence of CNTs entirely. The middle annular region (*R*_1_ < *r* ≤ *R*_2_) is modeled without further approximations. The parameters *R*_1_, *R*_2_, and *R*_3_ are adjustable to represent different CNT volume fractions.

Figure 1b illustrates the schematic of a single CNT unit. Here, we assume that each CNT has a radius denoted as *r*_*i*_ and that its diameter is a random variable obeying a log-normal distribution characterized by mean value *μ*_D_ and standard deviation *σ*_D_. We constructed a 3D schematic model of the Cu-CNT structure, as shown in Figure 1c; the length of the CNT is considered to be equal to the TSV height, represented as *L*. Additionally, we assume the total number of CNT samples, denoted as *N*, to be sufficiently large to ensure statistical validity.

We first derive the thermal resistance formula for the innermost layer. Since CNTs in this region are densely packed, the number of CNTs in the innermost layer, denoted as *n*, can be expressed as follows [16]:

(1) $$n=\frac{\pi (2R_{1}{)}^{2}}{4(2r_{i}+d{)}^{2}}$$

**Figure 2:**
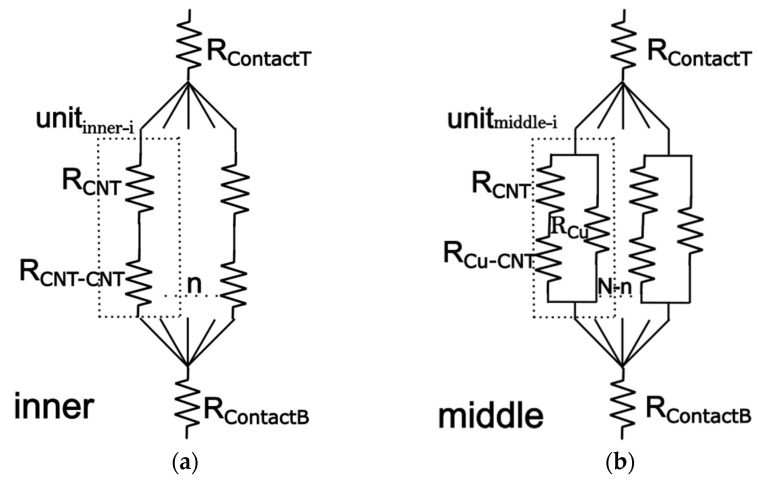
(**a**) Equivalent thermal resistance circuit of the inner Cu-CNT layer. (**b**) Equivalent thermal resistance circuit of the middle Cu-CNT layer. *R_middle-i_* represent the contact thermal resistance of the *i*-th CNT in the inner layer and middle layer. *R_contactT_* and *R_contactB_* denote the equivalent interfacial resistance between the TSV and the top/bottom connecting layers. *n* is the number of CNT units in the inner layer, and *N-n* the number of CNT units in the middle layer.

Figure 2a illustrates the thermal resistance model for the innermost layer. Given the close spacing between CNTs in this region, the thermal resistance arising from CNT-to-CNT contact must be considered [17]. Based on this consideration, the total thermal resistance for each individual CNT unit can be expressed as follows:

(2) $$R_{inner\text{-}i}=R_{CNT}\left(r_{i}\right)+R_{CNT\text{-}CNT}$$

Therefore, the total thermal resistance for the *n* parallel CNT units within the innermost layer is obtained as

(3) $$\frac{1}{R_{inner}}=\sum_{i=1}^{n}\frac{1}{R_{inner\text{-}i}}$$

Next, we derive the thermal resistance formula for the middle layer. Figure 2b shows the thermal resistance model for this layer, where CNTs are directly embedded within the Cu matrix. Here, we consider the interface thermal resistance between CNTs and the surrounding Cu matrix [17]. Based on the previous approximations, the number of CNTs in the middle layer is given by

(4) $$n_{2}=N-n$$

Using the thermal resistance model, we derive the total thermal resistance for each middle layer *R_middle-i_*. The total resistance for *n*_2_ units in parallel gives the total thermal resistance of the CNT units, *R_middle_*:

(5) $$R_{middle\text{-}i}=\frac{R_{Cu}R_{CNT}\left(r_{i}\right)}{R_{Cu}+R_{CNT}\left(r_{i}\right)}+R_{Cu\text{-}CNT}$$

(6) $$\frac{1}{R_{middle}}=\sum_{i=1}^{n_{2}}\frac{1}{R_{middle\text{-}i}}$$

Once the total thermal resistance is obtained, the thermal conductivity is calculated using the standard thermal conductivity formula:

(7) $$K=\frac{L}{R\cdot A}$$

(8) $$K_{eff1}=\frac{L}{R_{inner}\cdot \pi R_{1}^{2}}=\frac{L\cdot \sum_{i=1}^{n}\frac{1}{R_{inner\text{-}i}}}{\pi R_{1}^{2}}=\frac{L\cdot \sum_{i=1}^{n}\frac{1}{R_{CNT}\left(r_{i}\right)+R_{CNT\text{-}CNT}}}{\pi R_{1}^{2}}$$

It should be noted that the thermal resistance values provided in the parameters are area-specific thermal resistances. Therefore, they need to be converted into the corresponding actual thermal resistances in the subsequent calculation, which can be expressed as follows:

(9) $$R=\frac{R^{\prime }}{A}$$

After computing the total thermal conductivity of the CNT units, we apply the parallel thermal conductivity model to derive the effective thermal conductivity of the middle layer, *K*_*eff*2_:

(10) $$K_{eff2}=\varphi_{middle}K_{middle}+\left(1-\varphi_{middle}\right)K_{Cu}$$

(11) $$\varphi_{middle}=\frac{\sum_{i=1}^{n_{2}}A_{middle\text{-}i}}{\pi \left(R_{2}^{2}-R_{1}^{2}\right)}$$

(12) $$A_{middle\text{-}i}=(2r_{i}+d{)}^{2}$$

(13) $$K_{eff2}=\frac{\sum_{i=1}^{n_{2}}(2r_{i}+d{)}^{2}}{\pi \left(R_{2}^{2}-R_{1}^{2}\right)}\cdot \frac{L}{[\frac{R_{Cu}R_{CNT}\left(r_{i}\right)}{R_{Cu}+R_{CNT}\left(r_{i}\right)}+R_{Cu\text{-}CNT}]\cdot (2r_{i}+d{)}^{2}}+(1-\frac{\sum_{i=1}^{n_{2}}(2r_{i}+d{)}^{2}}{\pi \left(R_{2}^{2}-R_{1}^{2}\right)})K_{Cu}$$

According to our proposed model, the outermost TSV region is composed of Cu, with *R*_contactT_ and *R*_contactB_ representing the contact thermal resistance between the TSV and the upper and lower layers. At this point, we have derived the thermal conductivity formulas for the innermost, middle, and outermost layers. *K_eff_* denotes the overall effective thermal conductivity of the three-layer composite structure:

(14) $$K_{eff}=\frac{R_{1}^{2}}{R_{3}^{2}}\cdot K_{eff1}+\frac{R_{2}^{2}-R_{1}^{2}}{R_{3}^{2}}\cdot K_{eff2}+\frac{R_{3}^{2}-R_{2}^{2}}{R_{3}^{2}}\cdot K_{Cu}$$

### 2.3. Approximation of the Summation Formula

In the previously derived Equations (8) and (13), the variable *r*_*i*_ follows a log-normal distribution, making it difficult to obtain an analytical solution directly. To address this, we approximate the summation using the Law of Large Numbers (LLN) and the Gauss–Hermite quadrature method.

Gauss–Hermite quadrature is a form of Gaussian quadrature used to approximate integrals of the following type [18]:

(15) $$\int_{-\infty }^{+\infty }e^{-x^{2}}f\left(x\right)dx\approx \sum_{j=1}^{m}w_{j}f\left(x_{j}\right)$$

where the weight *w*_j_ is given by

(16) $$w_{j}=\frac{2^{m-1}m!\sqrt{\pi }}{m^{2}[H_{m-1}(x_{j}){]}^{2}}$$

The Hermite polynomial *H*_m_(*x*) is defined as

(17) $$H_{m}(x)=(-1{)}^{m}e^{x^{2}}\frac{d^{m}}{dx^{m}}e^{-x^{2}}$$

where *m* represents the number of sample points used, *x*_j_ are the roots of the Hermite polynomial *H*_m_(*x*), and *w*_j_ are the corresponding weight expressions. The accuracy of the approximation can be improved by increasing *m*.

According to the experimental characterization results, the CNT diameter *D* is assumed to follow a log-normal distribution, and thus

(18) $$ln\;D∼N(\mu_{D},\sigma_{D}^{2})$$

and the corresponding probability density function is given by

(19) $$f_{D}(D)=\frac{1}{D\sigma_{D}\sqrt{2\pi }}exp\left[-\frac{\left(ln\;D-\mu_{D}{)}^{2}\right.}{2\sigma_{D}^{2}}\right],D>0$$

Since the radius r is related to the diameter by

(20) D=2r

the logarithm of the radius can be written as

(21) ln r=ln D−ln 2

Therefore, ln *r* also follows a normal distribution, namely

(22) $$ln\;r∼N(\mu_{r},\sigma_{r}^{2})$$

with

(23) $$\mu_{r}=\mu_{D}-ln\;2,\sigma_{r}=\sigma_{D}$$

In this work, we primarily utilize the Gauss–Hermite quadrature method to compute expectation and variance. For a normally distributed random variable *y*, we can approximate the expectation of a function ℎ(*y*) using Gauss–Hermite quadrature as follows:

(24) $$y∼N\left(\mu ,\sigma^{2}\right)$$

the expectation of ℎ(*y*) can be written as

(25) $$E[h(y)]=\int_{-\infty }^{\infty }h(y)\frac{1}{\sqrt{2\pi }\sigma }exp\left[-\frac{\left(y-\mu {)}^{2}\right.}{2\sigma^{2}}\right]dy$$

By introducing the variable transformation

(26) $$x=\frac{y-\mu }{\sqrt{2}\sigma }$$

one obtains

(27) $$E[h(y)]=\frac{1}{\sqrt{\pi }}\int_{-\infty }^{\infty }h(\sqrt{2}\sigma x+\mu )e^{-x^{2}}dx$$

Then, by applying the Gauss–Hermite quadrature formula given in (15),

(28) $$E\left[h\left(y\right)\right]\approx \frac{1}{\sqrt{\pi }}\sum_{j=1}^{m}w_{j}h\left(\sqrt{2}\sigma x_{j}+\mu \right)$$

according to the LLN in probability theory, the sample mean of a large number of independent and identically distributed samples converges to the true mean:

(29) $$\frac{1}{N}\sum_{i=1}^{N}g\left(r_{i}\right)\to E\left[g\left(r_{i}\right)\right]$$

Combining (28) and (29) and substituting the result into (8) yields the expression for *K_eff_*_1_:

(30) $$K_{eff1}=\frac{L\cdot \sum_{i=1}^{n}\frac{1}{\frac{L}{k_{CNT}\pi r_{i}^{2}}+R_{CNT\text{-}CNT}}}{\pi R_{1}^{2}}\approx \frac{L\cdot \frac{n}{\sqrt{\pi }}\sum_{j=1}^{m}\frac{w_{j}}{\frac{L}{k_{CNT}\pi [exp\left(\sqrt{2}\sigma_{r}x_{j}+\mu_{r}\right){]}^{2}}+R_{CNT\text{-}CNT}}}{\pi R_{1}^{2}}$$

Here, since both *n* and *n*_2_ depend on the CNT radius *r_i_*, fully retaining the randomness of *r_i_* would make *n* and *n*_2_ random variables as well, thereby complicating the analytical derivation. Therefore, *r_i_* is approximated by its mean value *r_eq_* and both *n* and *n*_2_ are treated as constants.

(31) $$n\approx \frac{\pi (2R_{1}{)}^{2}}{4(2r_{eq}+d{)}^{2}}=\frac{\pi R_{1}^{2}}{{\left(2exp\left(\mu_{r}+\frac{{\sigma_{r}}^{2}}{2}\right)+d\right)}^{2}}$$

For brevity, we define $a_{j}$ as

(32) $$a_{j}=exp\left(\sqrt{2}\sigma_{r}x_{j}+\mu_{r}\right)$$

Combining (30) and (32) yields the expression for *K_eff_*_1_:

(33) $$K_{eff1}=\frac{L}{\pi R_{1}^{2}}\cdot \frac{n}{\sqrt{\pi }}\sum_{j=1}^{m}w_{j}\;\frac{1}{\frac{L}{k_{CNT}\pi a_{j}^{2}}+R_{CNT\text{-}CNT}}$$

Similarly, combining (13), (29), and (30) yields the expression for *K_eff_*_2_:

(34) $$k_{eff2}=\frac{L}{\pi \left(R_{2}^{2}-R_{1}^{2}\right)}\cdot \frac{n_{2}}{\sqrt{\pi }}\sum_{j=1}^{m}w_{j}\;\frac{1}{\frac{R_{Cu}\frac{L}{k_{CNT}\pi a_{j}^{2}}}{R_{Cu}+\frac{L}{k_{CNT}\pi a_{j}^{2}}}+R_{Cu\text{-}CNT}}+\left[1-\frac{n_{2}}{\pi \left(R_{2}^{2}-R_{1}^{2}\right)}\cdot \frac{1}{\sqrt{\pi }}\sum_{j=1}^{m}w_{j}\;{\left({2a}_{j}+d\right)}^{2}\right]K_{Cu}$$

By combining (30), (33) and (14), we obtain the final effective thermal conductivity of the TSV, *K_eff_*:

(35) $$K_{eff}\approx \frac{R_{1}^{2}}{R_{3}^{2}}\left[\frac{L}{\pi R_{1}^{2}}\cdot \frac{n}{\sqrt{\pi }}\sum_{j=1}^{m}w_{j}\;\frac{1}{\frac{L}{k_{CNT}\pi a_{j}^{2}}+R_{CNT-CNT}}\right]\;+\frac{R_{2}^{2}-R_{1}^{2}}{R_{3}^{2}}\left\{\frac{L}{\pi \left(R_{2}^{2}-R_{1}^{2}\right)}\cdot \frac{n_{2}}{\sqrt{\pi }}\sum_{j=1}^{m}w_{j}\;\frac{1}{\frac{R_{Cu}\frac{L}{k_{CNT}\pi a_{j}^{2}}}{R_{Cu}+\frac{L}{k_{CNT}\pi a_{j}^{2}}}+R_{Cu-CNT}}+\left[1-\frac{n_{2}}{\pi \left(R_{2}^{2}-R_{1}^{2}\right)}\cdot \frac{1}{\sqrt{\pi }}\sum_{j=1}^{m}w_{j}\;{\left({2a}_{j}+d\right)}^{2}\right]K_{Cu}\right\}+\frac{R_{3}^{2}-R_{2}^{2}}{R_{3}^{2}}K_{Cu}$$

**Figure 3:**
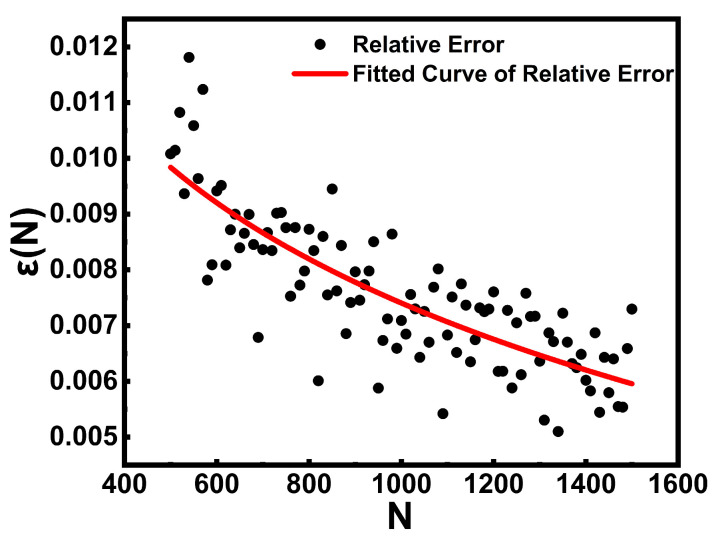
Scatter plot of the relative error *ε*(*N*) as a function of the CNT sample size *N*, together with the fitted trend curve obtained from nonlinear regression.

To verify the accuracy and validity of Equation (29), we performed a comparative analysis between the Gauss–Hermite (GH) quadrature method and MC simulations, treating the statistical average obtained from MC as the “reference solution.” The number of CNT samples *N* was swept from 500 to 1500 with a step of 10. For each *N*, the inner-layer effective thermal conductivity *K_eff_*_1_ was computed using both GH and MC. The relative error was defined as

(36) $$\varepsilon (N)=\frac{\left|g_{GH}(N)-g_{MC}(N)\right|}{g_{MC}(N)}$$

where g_MC_(*N*) denotes the MC-based estimate using *N* random samples, and g_GH_ (*N*) represents the approximate value calculated using the GH analytical method based on N random samples. To reduce statistical noise, 50 independent MC simulations were performed for each *N*, and their average value was used as the reference. The resulting relative errors were plotted as scatter points and fitted using a nonlinear regression curve, as shown in Figure 3.

The results show that the relative error decreases monotonically as *N* increases. When *N* > 1000, the error consistently remains below 10^−3^. This demonstrates that the GH quadrature method can accurately approximate the MC statistical average, confirming the validity of the approximation used in Equation (29). *N* = 1000 as the baseline parameter in this work provides a reliable balance between numerical accuracy and computational efficiency, further validating the feasibility and precision of the proposed analytical model.

### 2.4. Equivalent Interface Modeling and Process-Level Simplifications

To provide a complete and physically consistent description of the axial thermal path of the Cu-CNT TSV, several process-related structural effects are incorporated through effective interface resistances, while others are omitted due to their negligible contribution. The adopted treatment ensures that the model remains compact and general without relying on any specific packaging configuration.

To capture the thermal effects of packaging-level structures without relying on specific 3D IC implementations, effective contact thermal resistances *R*_contactT_ and *R*_contactB_ are introduced at the top and bottom of the TSV to represent the thermal discontinuities arising from the RDL, micro-bump layers, and the Cu-metal landing interface. These influences are compactly incorporated through

(37) $$R_{contactT}\approx R_{RDL}+R_{bump}+R_{contact\text{-}T}$$

(38) $$R_{contactB}\approx R_{RDL}+R_{bump}+R_{contact\text{-}B}$$

In this work, *K_total_* denotes the total thermal conductivity after additionally considering the interfacial contact resistances at the top and bottom layers:

(39) $$K_{total}=\frac{L}{L/K_{eff}+\pi R_{3}^{2}\left(R_{contactT}+R_{contactB}\right)}$$

In contrast, the depletion layer and the SiO_2_ insulation layer are not explicitly modeled because their influence on axial heat conduction is negligible. The depletion region formed in the silicon sidewall is extremely thin, affecting only the local electrical and high-frequency behavior [19] while contributing virtually nothing to the axial heat-flow path. The insulation layer primarily impacts radial heat spreading, and its axial thermal conductance is only about 0.06% of that of the Cu core under typical TSV dimensions. Therefore, neither effect is treated as an independent thermal resistance component, which helps maintain the simplicity and physical consistency of the proposed model.

## 3. Simulation Results and Discussion

The Cu-CNT TSV thermal analytical model proposed in this study is based on MC simulations and the Gaussian distribution approximation. To ensure the accuracy of the proposed model, we validate the simulation results by comparing them with experimental data from the existing literature. These experimental datasets primarily originate from previously published studies on thermal conductivity measurements of Cu-CNT composite materials. The simulation parameters are summarized in Table 1.

### 3.1. Sensitivity of Thermal Conductivity to Three-Layer Structural Parameters

**Figure 4:**
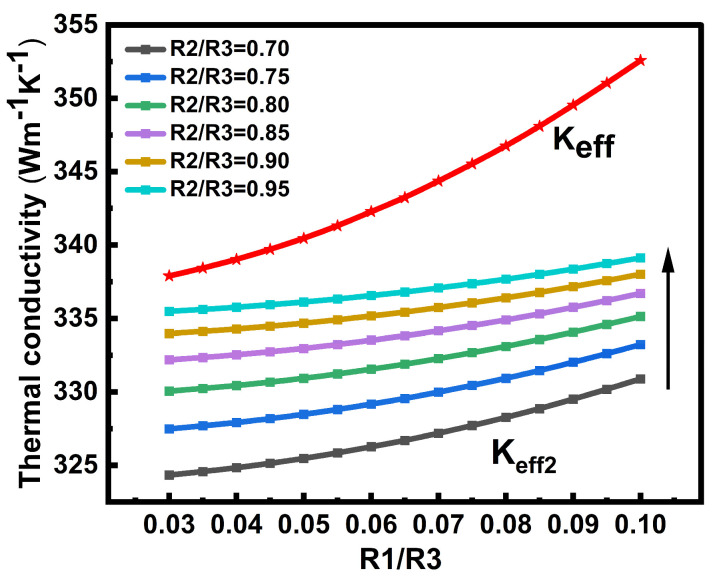
Influence of three-layer structural parameters on thermal conductivity. The arrow indicates the direction of increasing *R*_2_/*R*_3_.

To better explain the choice of *R*_1_, *R*_2_, *R*_3_ and their effects on thermal conductivity, we performed a sensitivity analysis on the normalized radii *R*_1_/*R*_3_ and *R*_2_/*R*_3_. As shown in Figure 4, the colored curves represent *K_eff_*_2_ under different *R*_2_/*R*_3_ values. For each fixed *R*_2_/*R*_3_, *K_eff_*_2_ increases monotonically with *R*_1_/*R*_3_. This is because, as *R*_1_ increases, the number of CNTs in the middle region decreases, which reduces the effect of the Cu-CNT interfacial thermal resistance and leads to a higher thermal conductivity. Meanwhile, *K_eff_*_2_ also increases with increasing *R*_2_/*R*_3_. This is because a larger *R*_2_ means a greater Cu contribution in the middle region, which improves its overall thermal conductivity.

The red curve represents the total thermal conductivity of the TSV, *K_eff_*. Only one red curve appears because, in the present model, the region between *R*_2_ and *R*_3_ is treated as pure Cu without CNTs. Therefore, changing *R*_2_/*R*_3_ does not affect *K_eff_*. The monotonic increase in the red curve with *R*_1_/*R*_3_ indicates that the increases in both inner and middle region thermal conductivity together enhance the overall thermal conductivity.

### 3.2. Analysis of Thermal Contact Resistance

**Figure 5:**
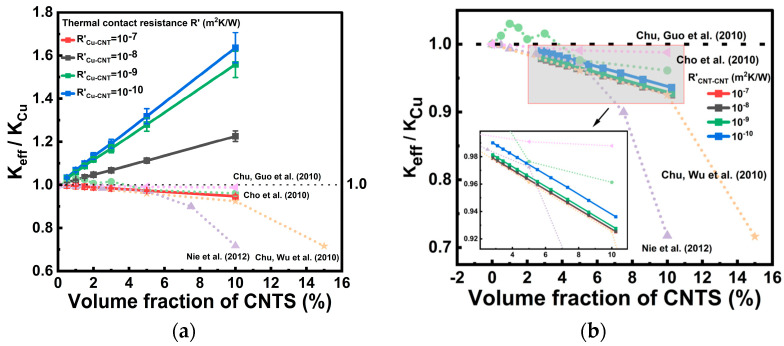
(**a**) Comparison between the simulated thermal conductivity of the Cu-CNT model with different Cu-CNT interfacial thermal resistances (*R’_CNT-CNT_* = 0) and the experimental data from [21,26,27,28]. (**b**) Comparison between the simulated thermal conductivity of the Cu-CNT model and different CNT-CNT interfacial thermal resistances (*R’_Cu-CNT_* = 10^−7^).

To identify the reasonable orders of magnitude of the interfacial thermal resistances, a two-step sensitivity analysis was carried out. First, in Figure 5a, *R’_CNT-CNT_* = 0 was set so that the effect of *R’_Cu-CNT_* on the overall thermal conductivity of the Cu-CNT composite could be examined independently. The reason for prioritizing the Cu-CNT interfacial resistance is that, in the present three-layer thermal resistance model, the Cu-CNT contacts are mainly associated with the middle region, whose cross-sectional area is significantly larger than that of the inner region.

The Cu-CNT interfacial thermal resistance *R*’_Cu-CNT_ was examined over the range of 10^−10^ to 10^−7^ m^2^K/W, which is consistent with typical values reported in the literature [17]. The results show that when *R’_Cu-CNT_* is low (10^−10^–10^−9^ m^2^K/W), *K_eff_/K_Cu_* increases markedly with increasing CNT volume fraction and reaches approximately 1.6–1.7 at a CNT volume fraction of about 10%, indicating that the intrinsically high thermal conductivity of CNTs can be effectively utilized under ideal interfacial conditions. In contrast, when *R’_Cu-CNT_* = 10^−7^ m^2^K/W, *K_eff_/K_Cu_* becomes slightly lower than 1 and gradually decreases to about 0.94–0.95 with increasing CNT volume fraction, it suggests that a large Cu-CNT interfacial thermal resistance can significantly suppress the thermal enhancement expected from CNT incorporation. Further comparison with the experimental data [21,26,27,28] shows that the measured values are overall much closer to the predicted curve for *R’_Cu-CNT_* = 10^−7^ m^2^K/W. This indicates that achieving an ultralow Cu-CNT interfacial thermal resistance remains challenging under current fabrication conditions, and thus *R’_Cu-CNT_* = 10^−7^ m^2^K/W can be regarded as a reasonable order-of-magnitude estimate for the subsequent analysis.

Figure 5b further fixes *R’_Cu-CNT_* = 10^−7^ m^2^K/W and investigates the effect of different orders of magnitude of *R’_CNT-CNT_* on the overall thermal conductivity. The results show that when *R’_CNT-CNT_* varies from 10^−10^ to 10^−7^ m^2^K/W, the variation in *K_eff_/K_Cu_* is relatively limited, while the overall trend still shows a gradual decrease with increasing CNT volume fraction, which is also consistent with the previous analysis. Compared with Figure 5a, this indicates that the influence of CNT-CNT contact thermal resistance on the overall thermal conductivity is weaker than that of the Cu-CNT interfacial thermal resistance. Even if *R’_CNT-CNT_* is reduced, it remains difficult to fundamentally reverse the thermally limited trend when *R’_Cu-CNT_* is relatively large. Therefore, the present results suggest that the Cu-CNT interfacial thermal resistance is the dominant factor affecting the thermal conductivity of Cu-CNT composite TSV within the present model. This further implies that, in practical fabrication, priority should be given to reducing the interfacial thermal resistance between Cu and CNTs in order to improve the thermal performance of Cu-CNT TSV.

### 3.3. Model Validation and Analysis

The simulation parameters are summarized in Table 1. Since the experimentally reported CNT diameters are mainly distributed within the range of 20–70 nm [21], a log-normal distribution is adopted in this work to approximately describe the CNT diameter distribution. The corresponding parameters are selected as *μ*_D_ = 3.7 and *σ*_D_ = 0.31 so that the resulting distribution can reasonably cover the experimentally reported diameter range. Meanwhile, the thermal conductivity values *K_CNT_* and *K_Cu_* in the model are also chosen to be consistent with the experimental conditions. As for the spatial distribution of CNTs, the experimental studies generally describe them as randomly distributed. On this basis, the present model further approximates the random dispersion characteristics of CNTs in the composite system by assuming that their positions follow a normal distribution. In this way, the model preserves analytical tractability while still reflecting, as much as possible, the actual structural features of the experimental samples. To further improve the consistency between the model parameters and the experimental system, the experimentally measured effective thermal conductivity values *K_eff_* at CNT volume fractions of 1% and 5% were selected as calibration points for fitting the interfacial thermal resistance parameters, because these two points can better represent the overall distribution trend of the data. By comparing the model predictions with the experimental data, the reasonable order of magnitude of *R’_CNT-CNT_* under the present experimental conditions was determined to be 10^−10^ m^2^K/W while that of *R’_Cu-CNT_* was determined to be 10^−7^ m^2^K/W.

**Figure 6:**
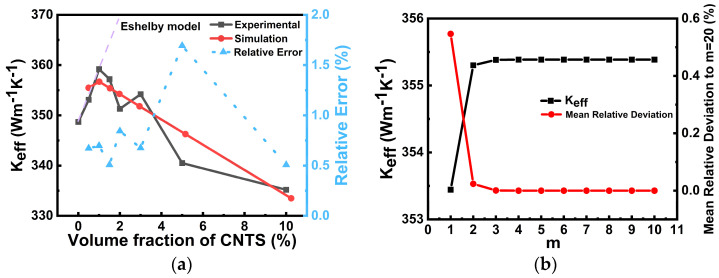
(**a**) Comparison of the effective thermal conductivity predicted by the present analytical model, the Eshelby model [21] and the experimental data [21] as a function of CNT volume fraction. (**b**) Effect of the Gauss–Hermite quadrature point number *m* on the calculated effective thermal conductivity for the present model at a CNT volume fraction of 1.5%.

As shown in Figure 6a, the simulation results obtained from the present model agree well with the experimental measurements over the investigated CNT volume-fraction range. Both the simulation and experimental data exhibit a non-monotonic variation trend: the effective thermal conductivity first increases slightly at low CNT contents and then gradually decreases as the CNT volume fraction further increases. In contrast, the Eshelby model predicts a monotonically increasing trend and significantly overestimates the thermal conductivity, especially at higher CNT contents. This is because the Eshelby equivalent inclusion model treats CNTs as ideal anisotropic inclusions embedded in a Cu matrix and does not explicitly account for the interfacial thermal resistance between CNTs and Cu [29], thus representing an idealized upper bound. The relative error between the present simulation and the experimental data remains low throughout the whole range and is generally below 2%, indicating that the proposed analytical model can provide a reliable prediction of the effective thermal conductivity of Cu-CNT composites.

From a physical point of view, the slight enhancement of thermal conductivity in the low-CNT regime can be attributed to the intrinsically high thermal conductivity of CNTs, which provides additional heat conduction paths in the Cu matrix. However, when the CNT concentration increases further, the adverse effects associated with CNT-Cu interfacial thermal resistance, CNT-CNT contact resistance, and possible structural complexities such as agglomeration and entanglement gradually become more significant [26]. As a result, the conductivity enhancement brought by CNT incorporation is weakened, and the overall effective thermal conductivity starts to decline. Therefore, the present model is able to capture the competition between the intrinsic thermal conductivity benefit of CNTs and the degradation caused by interfacial resistance and microstructural non-idealities more effectively than the Eshelby model. It should be noted that the present validation primarily supports the capability of the proposed model in predicting the effective thermal conductivity of Cu-CNT-filled TSV at the material-property level. While these results provide useful support for the reasonableness of the model, further device-level experimental investigation would still be needed to more fully validate the thermal behavior of actual TSV devices.

Figure 6b further presents the influence of the Gauss–Hermite quadrature point number m on the calculated effective thermal conductivity for the representative case of a CNT volume fraction of 1.5%. The results show that when *m* increases from 1 to 2, the predicted *K_eff_* changes noticeably, indicating that a very small number of quadrature points is insufficient to accurately describe the log-normal CNT diameter distribution. However, when *m* ≥ 3, the calculated *K_eff_* rapidly approaches a stable value, and the mean relative deviation with respect to the reference result at *m* = 20 decreases to nearly zero and remains almost unchanged thereafter. This demonstrates that the numerical integration converges quickly and that only a limited number of quadrature points is required to achieve stable and accurate predictions [18]. Therefore, the adopted Gauss–Hermite quadrature scheme is both efficient and reliable for the present analytical model.

### 3.4. Thermal Impact of the Parameters μ and σ

**Figure 7:**
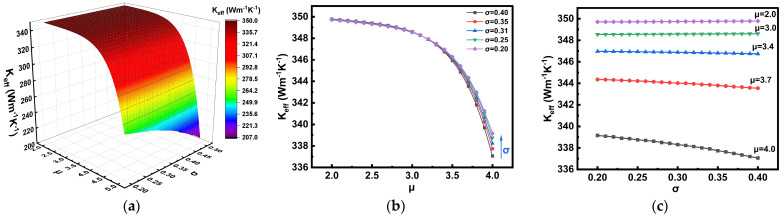
(**a**) Effective thermal conductivity *K_eff_* of Cu-CNT composites as a function of the log-normal distribution parameters *μ* and *σ*. The number of Gauss–Hermite quadrature points is set to *m* = 15 with *μ* ranging from 2 to 5 and *σ* ranging from 0.2 to 0.5, consistent with reported experimental ranges. (**b**) Variation in *K_eff_* with *μ* under different fixed *σ* values, where *σ* is set to five representative values: 0.2, 0.25, 0.31, 0.35, and 0.4. (**c**) Variation in *K_eff_* with *σ* under different fixed *μ* values, where *μ* is set to five representative values: 2.0, 3.0, 3.4, 3.7, and 4.0.

Figure 7 illustrates the effect of the CNT diameter log-normal distribution parameters, mean (*μ*) and standard deviation (*σ*), on the effective thermal conductivity (*K_eff_*) of Cu-CNT composites. As shown in Figure 7a, the three-dimensional surface plot reveals a strong dependence of *K_eff_* on both parameters. Specifically, a decrease in *μ* significantly enhances the effective thermal conductivity, while variations in σ have a relatively weaker impact, which is consistent with findings reported in the literature [30]. This behavior can be attributed to two main reasons: first, a smaller CNT diameter increases the number density of CNTs, particularly in the innermost region, thereby providing more efficient thermal conduction pathways. Second, a lower *σ* value indicates a more concentrated diameter distribution, resulting in more uniform spatial arrangement of CNTs and reduced interfacial thermal resistance, which marginally improves the overall heat transfer performance.

Figure 7b,c provides a more detailed quantitative analysis of these trends. Figure 7b shows the variation in *K_eff_* with *μ* under different fixed σ values. It is clearly observed that *K_eff_* decreases significantly as *μ* increases, particularly beyond *μ* = 3.5. Moreover, when *σ* is varied under fixed *μ*, the effect on *K_eff_* is relatively minor. For instance, as *σ* decreases from 0.4 to 0.2, the thermal conductivity improves by only 0.6%. Figure 7c further examines the dependence of *K_eff_* on σ under various fixed μ values. The results show that increasing σ leads to a continuous decline in *K_eff_*_,_ with the effect being more pronounced at larger *μ* values. This trend may be explained by the fact that, at larger diameters, the number of CNTs in the inner layer decreases while those in the middle layer increase, resulting in elevated Cu-CNT interfacial resistance and a reduction in the overall thermal conduction efficiency.

### 3.5. Thermal Impact of TSV Length in Cu-CNT

**Figure 8:**
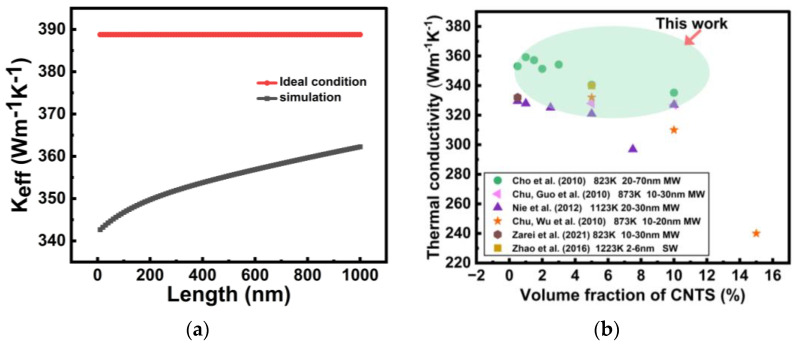
(**a**) Variation in the effective thermal conductivity *K_eff_* of the Cu-CNT composite with TSV length *L* under ideal and practical interfacial conditions. In the ideal condition, the interfacial thermal resistances are assumed to be zero. (**b**) Predicted thermal conductivity of Cu-CNT composites at different CNT volume fractions based on the proposed model. The green ellipse indicates the prediction range of this study, while the other data points represent experimental results from previous studies [21,26,27,28,31,32].

As shown in Figure 8a, the TSV length has a noticeable influence on the equivalent thermal conductivity *K_eff_* of the Cu-CNT composite structure under practical interfacial conditions. In the simulation, with *R’_CNT-CNT_* = 10^−10^ m^2^K/W and *R’_Cu-CNT_* = 10^−7^ m^2^K/W, *K_eff_* increases gradually as the TSV length *L* increases from 10 to 1000 nm.

The dependence of the effective thermal conductivity *K_eff_* on TSV length can be divided into two distinct regimes. In the short-length range of 10–200 nm, the simulated *K_eff_* increases relatively rapidly with increasing *L*. This indicates that interfacial thermal resistance contributes significantly to the total thermal resistance in short TSV, making the overall thermal conduction highly sensitive to length variation. As *L* increases, the relative effect of interfacial resistance is reduced, leading to a marked improvement in *K_eff_*. In the longer-length range of 200–1000 nm, *K_eff_* still increases with TSV length, but the growth rate becomes much smaller. This suggests that the relative contribution of interfacial thermal resistance has been substantially weakened, and the heat transport behavior gradually becomes dominated by the intrinsic thermal properties of the Cu-CNT composite. As a result, the effective thermal conductivity shows a tendency toward saturation at larger TSV lengths.

By contrast, under the ideal condition where *R’_CNT-CNT_* = *R’_Cu-CNT_* = 0, *K_eff_* remains nearly constant throughout the entire length range. This indicates that, in the absence of interfacial thermal resistance, heat transport is dominated by the intrinsic thermal properties of the constituent materials, and the equivalent thermal conductivity is therefore essentially independent of geometric length. It is also worth noting that the simulated *K_eff_* remains consistently lower than the ideal value throughout the entire range, indicating that non-ideal interfacial effects are still a key factor limiting the thermal transport performance of Cu-CNT composite TSVs.

### 3.6. Predictive Range of the Cu-CNT TSV Model

Figure 8b presents the predictive range of thermal conductivity derived from the Cu-CNT TSV model proposed in this study (represented by the green elliptical region) and compares it with the experimental measurements reported in the literature. The x-axis represents the CNT volume fraction, while the y-axis denotes the thermal conductivity. Since the parameters *R*_1_, *R*_2_, and *R*_3_ in the model are adjustable, the predicted thermal conductivity is not a single fixed value, but rather distributed within a specific range. From Figure 8b, it can be observed that the predicted thermal conductivity range of our model exhibits strong consistency with the experimental data obtained from various research groups. This indicates that the proposed model can reasonably predict the thermal conductivity of Cu-CNT composites with varying CNT volume fractions, providing useful guidance for future practical fabrication processes.

It is worth noting that some literature-reported data deviate from our predicted range. These discrepancies may be attributed to variations in CNT fabrication methods, interfacial treatment processes, and other experimental factors, leading to significant differences in thermal conductivity even at similar CNT volume fractions. In future work, further optimization of model parameters could enhance the generalizability and predictive accuracy, providing a more precise theoretical foundation for process optimization in practical applications.

## 4. Three Backside Metal Layers nTSV Structure

To more intuitively demonstrate the thermal characteristics of Cu-CNT TSVs, we further built a nano-TSV (nTSV) model with three backside metal (BSM) layers in COMSOL Multiphysics (version 6.1, COMSOL AB, Stockholm, Sweden).

### 4.1. Simulation Parameter Setup

**Figure 9:**
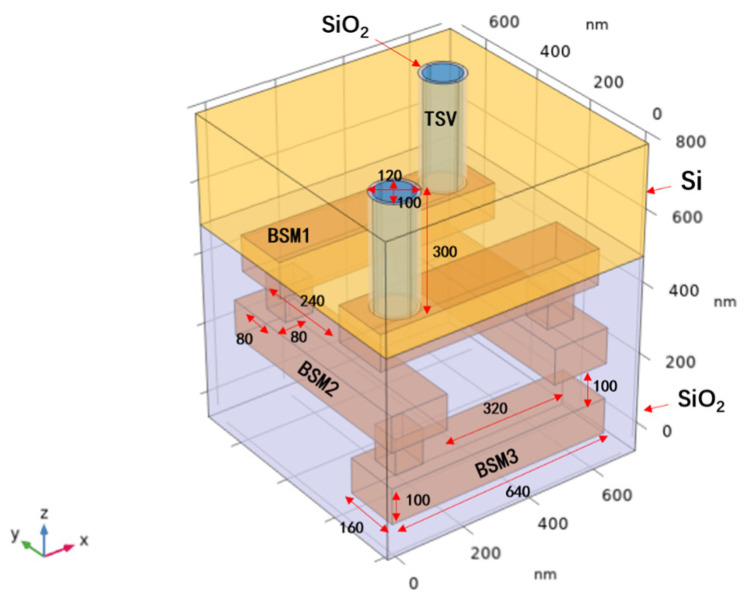
Three-BSM-layer nTSV structure. Schematic of the proposed structure with key dimensions labeled. The BSM region is embedded in SiO_2_ dielectric fill, while the nTSV is surrounded by a SiO_2_ oxide liner.

As shown in Figure 9, the backside metal layers are made of Cu, while the nTSV is alternatively filled with Ru, Cu, and Cu-CNT composite for comparison. The structure consists of three backside metal layers, and the nTSV penetrates the silicon substrate to emulate the backside power delivery network (BSPDN) configuration. In addition, the nTSV is surrounded by a SiO_2_ liner, which is treated as a barrier layer. The overall geometry and dimensions of this structure follow the design reported by Chen et al. [33].

A coupled electro-thermal simulation was carried out in COMSOL Multiphysics using the electromagnetic heating multiphysics interface. For the thermal setup, the top surfaces of the two TSVs were treated as heat sources by applying a general inward heat flux of *Q*_0_ = 1 × 10^6^ W/m^2^ [34], representing a relatively high heat flux operating condition for evaluating the thermal dissipation capability of the structure. Meanwhile, convective cooling was imposed at the bottom surface with a heat transfer coefficient of *h* = 500 W/(m^2^K) [35], representing a strong air-cooling condition. For the electrical setup, a potential of 0.5 μV [36] was applied at the top of the structure to represent the voltage drop in the model interconnect, while the opposite end was grounded to complete the current return path. The other boundaries were kept electrically and thermally independent. The initial temperature for the calculation was set to 293.15 K. The COMSOL simulation parameters are summarized in Table 2, where the thermal conductivity of Cu-CNT is obtained from our previously derived analytical model.

More specifically, to establish the link between the analytical model and the device-level COMSOL simulation, the characteristic dimensions adopted in the analytical calculation were chosen to be consistent with the nTSV geometry used in the COMSOL structure. Considering that the present analytical model shows better agreement with reported experimental data in the low CNT concentration regime, the CNT content in the Cu-CNT filling was controlled to remain below 10%. Under this constraint, the CNT diameter was assumed to follow a log-normal distribution with *μ*_D_ = 1.0 and *σ*_D_ = 0.31, and the total number of CNTs was set to *N* = 100. In addition, the interfacial thermal resistance between adjacent CNTs was taken as *R’_CNT-CNT_* = 10^−10^ m^2^K/W, while the Cu-CNT interfacial thermal resistance was set to *R’_Cu-CNT_* = 10^−8^ m^2^K/W. By substituting the above parameter values into the analytical model, the calculated thermal conductivity of the Cu-CNT TSV is *K_total_* = 339.919 W/(m·K).

### 4.2. COMSOL Simulation Results and Discussion

**Figure 10:**
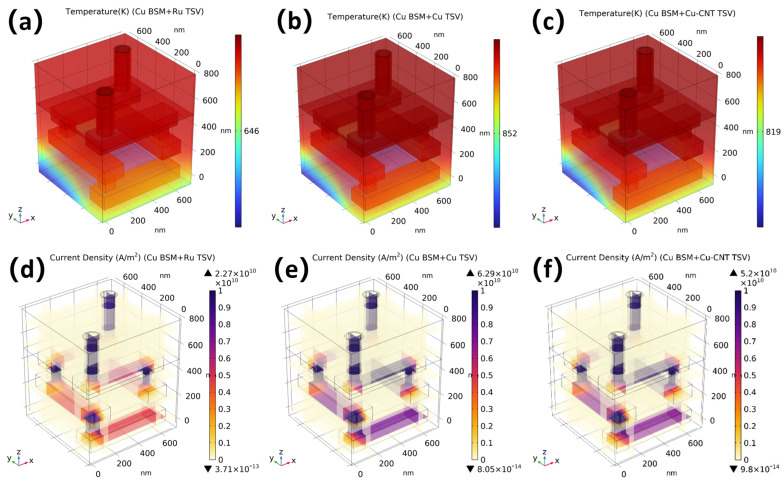
The temperature profiles and current density distributions under different TSV filling materials, with Cu used as the BSM. Temperature profiles of three-BSM-layer nTSV based on (**a**) Cu, (**b**) Ru and (**c**) Cu-CNT. Current density profiles of three-BSM-layer nTSV based on (**d**) Cu, (**e**) Ru and (**f**) Cu-CNT.

Figure 10 compares the electro-thermal responses of the Cu backside metal structure when the nTSVs are filled with Cu, Ru, and Cu-CNT composite, respectively. As shown by the temperature fields in Figure 10a–c, all three cases exhibit a consistent vertical temperature gradient: the hot region is mainly localized in the nTSV and its adjacent interconnects, whereas the bottom region remains the coolest. This indicates that the nTSV serves as one of the dominant heat-flow paths between the stacked tiers. In addition, the front-side nTSV is generally hotter than the rear-side nTSV, which can be attributed to the presence of an extra lateral heat-spreading path near the rear nTSV provided by the Cu BSM, thereby enhancing the local heat dissipation capability.

In terms of hotspot temperature, the filling material has a significant influence on the electro-thermal behavior. Among the three cases, the Cu TSV exhibits the highest peak temperature of 852 K, whereas the Ru TSV shows the lowest value of 646 K. The Cu-CNT TSV gives an intermediate peak temperature of 819 K. Compared with the Cu TSV, the Cu-CNT TSV reduces the peak temperature by about 3.9%, indicating that the Cu-CNT composite filling can alleviate the hotspot to some extent under the present parameter setting. By contrast, the Ru TSV achieves a much more pronounced temperature reduction of about 24.2% relative to the Cu TSV, indicating a better heat dissipation performance.

A similar trend is observed in the current density distributions shown in Figure 10d–f. The Cu TSV exhibits the highest peak current density, reaching 6.29 × 10^10^ A/m^2^, together with pronounced current crowding near the contact regions. The Ru TSV shows the lowest peak current density of 2.27 × 10^10^ A/m^2^, while the Cu-CNT TSV reaches 5.20 × 10^10^ A/m^2^, which is about 17.3% lower than that of the Cu TSV but still significantly higher than that of the Ru TSV. This indicates that, under the current electrical parameter setting, the Cu–CNT structure can only partially mitigate the local current concentration, whereas the Ru TSV suppresses current crowding and the associated Joule heating more effectively due to its lower overall current level.

Overall, the updated results suggest that, under the present parameter set, the electro-thermal performance of the Cu-CNT nTSV is improved compared with that of the conventional Cu TSV, but it does not surpass that of the Ru TSV.

## 5. Conclusions

In this study, we develop an analytical model for estimating the effective thermal conductivity of a Cu-CNT-filled TSV by combining Monte Carlo simulation, Gaussian distribution-based approximation, and interfacial thermal resistance effects. Through a two-step sensitivity analysis, the interfacial thermal resistance of Cu-CNT was estimated to be on the order of *R’_Cu-CNT_* = 10^−7^ m^2^K/W. Through calibration against previously reported effective thermal conductivity data of Cu-CNT composites, the analytical model was shown to achieve high predictive accuracy when the CNT volume fraction is below 10%, with an overall error of less than 2%. It should be emphasized that the present validation primarily supports the model capability in predicting effective thermal conductivity at the material level, rather than constituting a direct TSV-level experimental validation of full thermal behavior. The proposed analytical model is therefore most suitable for the low-CNT-volume-fraction regime, which is also consistent with the parameter range commonly encountered in current fabrication processes. We further investigate the effects of CNT diameter distribution parameters and TSV length on the effective thermal conductivity. The results indicate that reducing the mean CNT diameter *μ* significantly enhances the thermal conductivity, particularly when *μ* < 3.5, whereas decreasing *σ* leads to only a limited improvement of about 0.6%. In addition, to demonstrate the device-level applicability of the extracted effective thermal conductivity, coupled electro-thermal simulations were further carried out for the three-BSM-layer nTSV structure. The results show that, under the present parameter setting, the Cu-CNT nTSV provides a moderate improvement over the conventional Cu nTSV, with the peak temperature decreasing from 852 K to 819 K and the peak current density decreasing from 6.29 × 10^10^ A/m^2^ to 5.20 × 10^10^ A/m^2^. However, the Ru nTSV still exhibits the best overall electro-thermal performance in the current comparison. Overall, this work establishes an analytical framework for predicting the effective thermal conductivity of Cu-CNT TSVs and for supplying material-level thermal inputs to device-level electro-thermal simulations, while also indicating that the performance of Cu-CNT TSVs remains highly sensitive to material parameters, interfacial resistances, and process conditions.

## Figures and Tables

**Table 1:** Summary of modeling parameters.

Symbol	Quantity	Description
Geometric parameters		
*μ*_D_	3.7 [13]	Mean of the log-normal distribution for CNT diameters
*σ*_D_	0.31 [13]	Log-normal standard deviation of CNT diameters
*N*	1000	Number of carbon nanotubes
*d*	0.34 nm [16]	Minimum distance between two CNTs
*L*	10–1000 nm	Length of the TSV
Material properties		
*K_CNT_*	3000 W/(m·K) [20]	Thermal conductivity of MWCNT
*K* *_Cu_*	348.7 W/(m·K) [21]	Thermal conductivity of Cu
Interfacial thermal resistances		
*R* *’_Cu_*_-*CNT*_	10^−10^–10^−7^ m^2^K/W [17]	Thermal contact resistance of Cu-CNT
*R* *’* *_CNT_*_-*CNT*_	10^−10^–10^−7^ m^2^K/W [22]	Thermal contact resistance of CNT-CNT
Packaging-level thermal resistances		
*R* *’* *_contact_*_-*T*_	10^−7^ m^2^K/W [17,23]	Effective thermal contact resistance at the TSV top interface
*R* *’* *_contact-B_*	10^−7^ m^2^K/W [17,23]	Effective thermal contact resistance at the TSV bottom interface
*R* *’* *_RDL_*	10^−7^ m^2^K/W [24]	Thermal resistance associated with the redistribution layer
*R* *’* *_BUMP_*	10^−7^–10^−6^ m^2^K/W [25]	Thermal resistance of the micro-bump and UBM stack

**Table 2:** Summary of simulation parameters in COMSOL.

Symbol	Quantity	Description
*K_C_* *_u_*	348.7 W/(m·K) [17]	Thermal conductivity of Cu
*K* *_Ru_*	125 W/(m·K) [1]	Thermal conductivity of Ru
*K* *_Cu-CNT_*	339.919 W/(m·K)	Calculated thermal conductivity of Cu-CNT based on the proposed model
*C_p_Cu_*	385 J/(kg·K)	Specific heat capacity of Cu
*C_p_Ru_*	238 J/(kg·K)	Specific heat capacity of Ru
*C_p_Cu-CNT_*	380 J/(kg·K) [9]	Specific heat capacity of Cu-CNT
*σ_Cu_*	4 × 10^7^ S/m [33]	Electrical conductivity of Cu
*σ_Ru_*	1.14 × 10^7^ S/m [1]	Electrical conductivity of Ru
*σ_Cu-CNT_*	3.2 × 10^7^ S/m [37]	Electrical conductivity of Cu-CNT

## Data Availability

Data will be made available upon request.
